# More than one shade of pink as a marker of early amelanotic/hypomelanotic melanoma

**DOI:** 10.1111/1346-8138.17200

**Published:** 2024-03-25

**Authors:** M. A. Pizzichetta, P. Corsetti, I. Stanganelli, G. Ghigliotti, S. Cavicchini, V. De Giorgi, R. Bono, S. Astorino, S. Ribero, G. Argenziano, Alaibac, J. Polesel, the Italian Melanoma Intergroup (IMI) and the Società Italiana di Dermatologia Chirurgica, Oncologica, Correttiva ed Estetica (SIDCO)

**Affiliations:** ^1^ Department of Dermatology University of Trieste Trieste Italy; ^2^ Department of Medical Oncology Centro di Riferimento Oncologico di Aviano (CRO) IRCCS Aviano Italy; ^3^ Skin Cancer Unit Istituto Tumori Romagna (IRST) Meldola Italy; ^4^ Department of Dermatology University of Parma Parma Italy; ^5^ Private Dermatologist Genova Italy; ^6^ Private Dermatologist Milan Italy; ^7^ Department of Dermatology University of Florence Florence Italy; ^8^ Private Dermatologist Roma Italy; ^9^ Division of Dermatology Celio Hospital Rome Italy; ^10^ Department Medical Sciences, Dermatologic Clinic University of Torino Turin Italy; ^11^ Dermatology Unit Second University of Naples Naples Italy; ^12^ Department of Dermatology University of Padova Padova Italy; ^13^ Cancer Epidemiology Unit Centro di Riferimento Oncologico di Aviano (CRO) IRCCS Aviano Italy

**Keywords:** amelanotic melanoma, benign melanocytic lesion, non‐melanocytic lesion

## Abstract

Amelanotic/hypomelanotic melanoma (AHM) may be difficult to diagnose because of a lack of pigmentation. To evaluate whether dermoscopy can be useful for the diagnosis of early AHM, 133 digital dermoscopic images of lesions histopathologically diagnosed as amelanotic/hypomelanotic superficial spreading melanoma with ≤1 mm thickness (AHSSMs) (*n* = 27), amelanotic/hypomelanotic non‐melanocytic lesions (AHNMLs) (e.g., seborrhoeic keratosis and basal cell carcinoma) (*n* = 79), and amelanotic/hypomelanotic benign melanocytic lesions (AHBMLs) (e.g., compound and dermal nevi) (*n* = 27), were dermoscopically assessed by three blinded dermatologists. Using multivariate analysis, we found a significantly increased risk of diagnosing AHSSM versus AHNML and AHBML when the lesion was characterized by the presence of more than one shade of pink (odds ratio [OR] 37.11), irregular dots/globules (OR 23.73), asymmetric pigmentation (OR 8.85), and structureless pattern (OR 7.33). In conclusion, dermoscopy may improve early AHM detection, discriminating AHSSM from amelanotic/hypomelanotic non melanoma lesions.

## 1 INTRODUCTION

Amelanotic/hypomelanotic melanoma (AHM) is a subtype of melanoma that includes both hypomelanotic (partially pigmented and light colored) and amelanotic melanoma (AM) with no melanin pigmentation.^1^ The diagnosis of AHM may be very difficult because of the absence of pigmentation and presence of symmetry.^2^ Few studies have described the dermoscopic features of early AHM in detail.^1^, ^2^, ^3^ In this retrospective study, 133 amelanotic/hypomelanotic skin lesions were evaluated dermoscopically to identify predictive features of amelanotic/hypomelanotic superficial spreading melanoma (AHSSM) of ≤1 mm thickness.

## 2 PATIENTS AND METHODS

We collected 133 cases of histopathologically confirmed AHSSMs, amelanotic/hypomelanotic benign melanocytic lesions (AHBMLs) (e.g., compound and dermal nevus), and amelanotic/hypomelanotic non‐melanocytic lesions (AHNMLs) (e.g. seborrhoeic keratosis and basal cell carcinoma) from eight participating centers between January 2007 and December 2011. This series was partially derived from our previous study.^2^

In this study only amelanotic/hypomelanotic lesions were considered, including amelanotic, having no melanin pigmentation (i.e., tan, dark brown, blue, gray, or black) and hypomelanotic melanomas. The latter comprise both partially pigmented lesions with a melanin pigmentation area ≤25% and light‐colored melanoma with a faint brown or light gray‐blue pigmentation.^1^ All cases were examined to assess the presence or absence of specific criteria by a panel of three blinded observers. The evaluation of the dermoscopic criteria was made when 3/3 or 2/3 observers agreed.

To identify the relevant factors associated with the diagnosis of AHSMM versus AHBML plus AHNML, univariate and multivariate analyses were used to compute odds ratios (ORs) and their corresponding 95% confidence intervals (CIs). Statistical significance was claimed for *p* values of ≤0.05 (two‐sided).

The approval by the Board of Ethics is waived for retrospective studies in accordance with the Italian research regulations (D.Lgs. 101/2018, art. 8), as the patients give their consent to the use of clinical data for research purposes, including publication of photographic material, at hospitalization.

## 3 RESULTS

The study consisted of 133 amelanotic/hypomelanotic skin lesions in 133 patients. The AHSSMs group (*n* = 27) included five cases of in situ melanoma and 22 cases of invasive melanoma with a median Breslow thickness of 0.47 mm (range, 0.20–1.00 mm).

The information on amelanotic or hypomelanotic type was available for 21 cases. Among these cases, 19 out 21 cases (90%) were AM.

The comparison group included 79 AHNMLs (51 basal cell carcinomas and 28 seborrhoeic keratosis) and 27 AHBMLs (compound/dermal nevi).

The study comprised 65 males and 68 females with a median age of 57 years (range, 24–84 years). No differences by sex emerged across the study groups, however, AHBML patients were younger than AHSSM and AHNML patients (median age 39 years vs 61 years and 63 years respectively). The back was the most frequent lesion localization area in all study groups.

Table 1 shows the dermoscopic features that were significantly associated with the diagnosis of AHSSM versus AHBML/AHNML in the univariate analysis. Among them, at the multivariable analysis, four dermoscopic features were significantly and independently associated with AHSSM, namely more than one shade of pink (OR 37.11, 95% CI 6.98–197.37), irregular dots/globules (OR 23.73, 95% CI 2.86–186.85), asymmetric pigmentation pattern (OR 8.85, 95% CI 1.24–63.10), and a structureless pattern (OR 7.33, 95% CI 1.21–44.61). Notably, the first three features were highly frequent in AHSMM, reported in more than 75% of cases.

**TABLE 1:** Univariate and multivariate analyses of positive dermoscopic features of AHSSM versus AHBML+AHNML.

Positive features	AHSSM (*n* = 27)	AHBML+ AHNML (*n* = 106)	OR (95% CI)	
			Univariate	Multivariate^a^
	*n* (%)	*n* (%)		
More than one shade of pink	21 (77.8)	5 (4.7)	70.70 (19.73–253.40) *p* < 0.0001	37.11 (6.98–197.37) *p* < 0.0001
Irregular dots/globules	25 (92.6)	27 (25.5)	36.57 (8.12–164.75) *p* < 0.0001	23.73 (2.86–186.85) *p* = 0.0034
Asymmetric pigmentation pattern	25 (92.6)	44 (41.5)	17.61 (3.97–78.25) *p* = 0.0002	8.85 (1.24–63.10) *p* = 0.0296
Atypical network	9 (33.3)	5 (4.7)	10.10 (3.03–33.63) *p* = 0.0002	
Structureless pattern	11 (40.7)	10 (9.4)	6.60 (2.41–18.06) *p* = 0.0002	7.33 (1.21–44.61) *p* = 0.0306
Multicomponent pattern	9 (33.3)	1 (0.9)	52.48 (6.27–439.53) *p* = 0.0003	
Irregular blotches	7 (25.9)	4 (3.8)	8.93 (2.39–33.37) *p* = 0.0011	
Irregular streaks	6 (22.2)	1 (0.9)	30.00 (3.43–262.28) *p* = 0.0021	
Irregular depigmentation	16 (59.3)	29 (27.4)	3.86 (1.60–9.30) *p* = 0.0026	
Regression structures	10 (37.0)	13 (12.3)	4.21 (1.59–11.14) *p* = 0.0038	
Shiny white structures	10 (37.0)	15 (14.2)	3.57 (1.38–9.26) *p* = 0.0089	
Asymmetric shape	11 (40.7)	21 (19.8)	2.78 (1.13–6.87) *p* = 0.0265	
Peripheral light brown structureless areas	4 (14.8)	4 (3.8)	4.44 (1.03–19.06) *p* = 0.0452	

Abbreviations: AHBML, amelanotic/hypomelanotic benign melanocytic lesions; AHNML, amelanotic/hypomelanotic nonmelanocytic lesions; AHSSM, amelanotic/hypomelanotic superficial spreading melanoma with ≤1 mm thickness; CI, confidence interval; OR, odds ratio.

^a^ Estimated from stepwise unconditional logistic regression model including all significant features in the univariate analysis.

## 4 DISCUSSION

**FIGURE 1:**
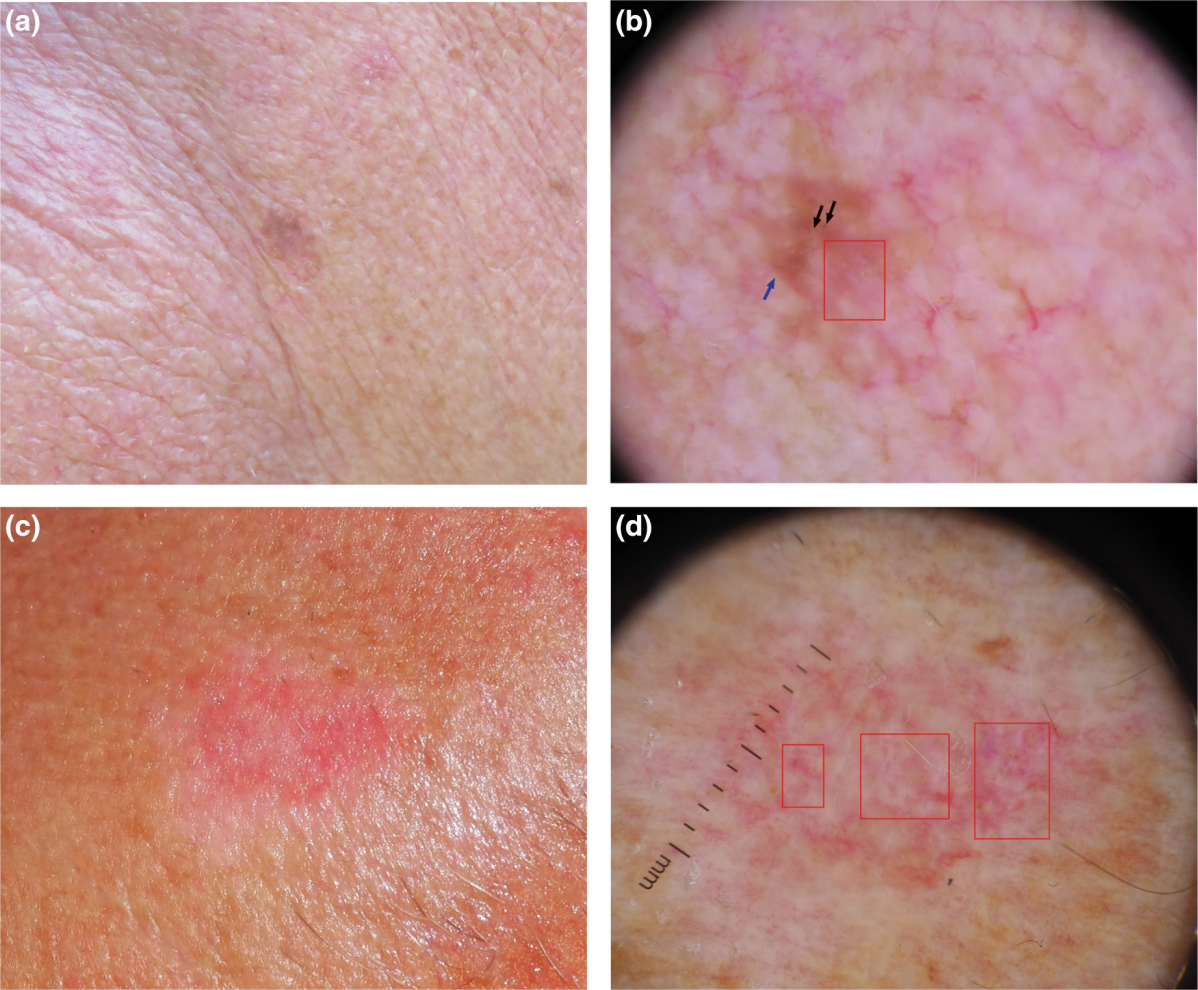
(a, b) Clinical and dermoscopic image of hypomelanotic melanoma in situ on the neck of an 80 year old man. (a) In the clinical image a pink‐brownish asymmetrical macule with irregular borders can be observed. (b) Dermoscopically, the melanoma reveals a structureless pattern, more than one shade of pink (square) and a peripheral light brown pigmentation (blue arrow) with foci of irregular brown dots/globules, (black arrow). (c, d) Clinical and dermoscopic images of amelanotic superficial spreading melanoma with a Breslow thickness of 0.2 mm on the neck of a 61‐year‐old man. (c) In the clinical image a pink reddish macule with irregular borders surrounded by a white asymmetric halo can be seen. (d) The dermoscopic image of the same lesion shows a structureless pattern, ill‐defined areas of a milky red color with more than one shade of pink throughout the lesion, without clearly visible vascularity (squares), (original magnification 10×).

**FIGURE 2:**
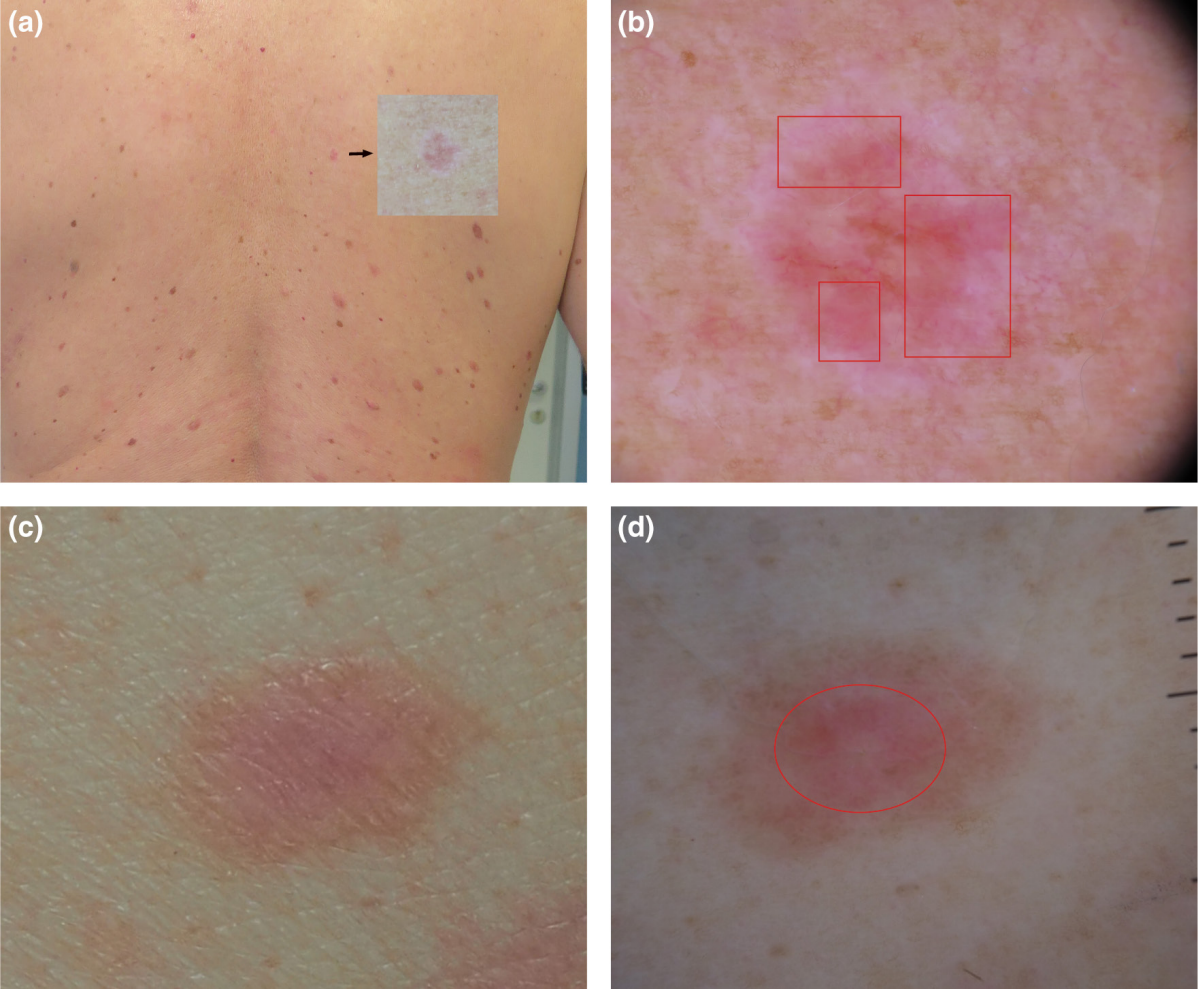
(a, b) Clinical and dermoscopic image of amelanotic melanoma in situ on the back of a 50 year‐old man. (a) Magnified detail (arrow) of a pink macule, surrounded by whitish irregular halo, on the back of a patient having multiple pink lesions, can be seen. (b) The dermoscopic image shows an overall structureless pattern consisting of ill‐defined areas of milky red background presenting more than one shade of pink throughout the lesion (squares), that allows the identification of the “little red riding hood sign”; the more innocent lesion could clinically look benign from a distance but not when seen close up with dermoscopy. (c, d) Clinical and dermoscopic images of a compound nevus on the back of a 40‐year‐old man. (c) In the clinical image a pink symmetric papule can be observed. (d) The dermoscopic image of the same nevus shows a pink colored center surrounded by a peripheral light brown pigmentation not visible to the naked eye (original magnification 10×). The presence of more than one shade of pink throughout the lesion can help in discriminating early amelanotic melanoma from pink melanocytic nevus in which the pink color is located more frequently in the center, and the silhouette of the lesion can be discerned the at edge.

Many features, such as atypical network, multicomponent pattern, irregular blotches, irregular streaks, irregular depigmentation, regression structures, shiny white structures, asymmetric shape, and peripheral light brown structureless areas, were significantly more frequent in AHSSM compared with AHBML plus AHNML in the univariate analysis. The most striking results of our study were that the only significant features discriminating AHSSM from AHBML plus AHNML in the multivariable analysis were more than one shade of pink, irregular dots/globules, asymmetric pigmentation, and a structureless pattern. More than one shade of pink, also known as pink veil or milky red areas, probably correspond to areas with increased vascular volume.^1^ Neoangiogenesis is a dynamic process occurring during the rapid proliferation of melanocytes that determine an increase of oxygen and nutrient demands leading to hypoxia.^4^ Neoangiogenesis following the initial disarranged melanoma growth could satisfy increased requirements, playing an essential role in melanoma development.^4^ In fact, melanoma seems to contain more blood vessels than dysplastic nevi and these latter contain more blood vessels than common nevi.^5^ Moreover, a high density vascular network seems to be associated with the evolution of atypical intraepidermal melanocytic to melanoma in situ.^6^ In our study, AHSSM showed a significantly greater frequency of more than one shade of pink and this vascular‐related feature could be considered a marker of early AHM (Figures 1b,d and 2b). In fact, milky red areas were significantly associated with an augmented risk of amelanotic/hypomelanotic lentigo maligna.^7^

In our study, a structureless or non‐specific pattern was significantly associated with AHSSM. This pattern, correlated with absence or reduced amounts of the features seen in AHM (Figures 1d and 2b), has already been significantly associated with melanoma in situ.^8^

In agreement with our previous study, AHSSM showed a significantly greater frequency of irregular dots/globules and asymmetric pigmentation, (Figure 1b).^3^

Histopathologically, globules correspond to nests of melanocytes at the dermo‐epidermal junction or dermis, and dots with aggregates of melanocytes or melanin granules in the dermis.^9^ The pigmentation correlates aggregates of melanin in one or more layers of the epidermis and upper dermis.^9^

Although AM show no melanin pigmentation to the naked eye, melanosomes in various stages of melanization and melanin granules can be found on histopathological examination.^10^ Dermoscopy, thanks to visualization of criteria reflecting pigmentation not visible to the naked eye, improves the diagnosis of AHM, (Figure 1b).

The dermoscopic diagnosis for melanoma completely devoid of pigment, relies on the analysis of vascular related features and vascular structures. The morphology of vessels depends on the thickness of the melanoma. Early AHM is characterized by predominant dotted vessels, often orderly arranged within ill‐defined areas of milky red background, presenting more than one shade of pink.^11^ The combination of dotted and linear irregular vessels may be an indicator for intermediate thickness (1–2 mm) while thick AHM (>2 mm thickness) exhibits a polymorphous vascular pattern consisting of hairpin, linear coiled, linear helical, and arborizing vessels.^11^, ^12^ However, in the present study, we focused on the dermoscopic features discriminating early AHM from no‐melanoma lesions. In our study, dotted vessels were not significantly associated with AHSSM in comparison with non‐melanoma lesions. Conversely, we found in AHSSM a significantly greater frequency of more than one shade of pink without clearly visible vascularity on dermoscopy (Figures 1d and 2b). The presence of a prevalent milky red color, covering more than 40% of the lesion surface, has already been considered a suspicious criterion that may assist in the differentiation between benign and suspicious pink lesions.^13^

The location of the pink color in a dermoscopic image may also be a clue to the dermoscopic diagnosis of melanoma. Pink throughout or in the periphery of the lesion has been reported more frequently in melanomas (Figure 2b), differently from benign melanocytic lesions where the pink color is located more frequently in the center of the lesion (Figure 2d).^14^

However, for the diagnosis of difficult, amelanotic lesions the optimal approach may be the combination of clinical information with total body dermoscopic examination, searching for the so called “little red riding hood sign”. The more innocent lesion could clinically look benign from a distance (Figure 2a) but not when seen close up with dermoscopy, thanks to its ability to visualize more than one shade of pink (Figure 2b).

Clinical information and the consideration of high‐risk features for AHM^15^ play an important role in the early diagnosis of AHM. The presence of a pink lesion in patients with red hair, fair skin, many freckles, few nevi, and a history of actinic keratoses and non‐melanoma skin cancer increases the index of suspicion that such a lesion could be a melanoma. In conclusion, because dermoscopy uses features reflecting increased vascular volume (more than one shade of pink) and pigmentation (irregular dots/globules and asymmetric pigmentation pattern), it may improve AHSSM detection. The early AHM stage may be characterized only by more than one shade of pink, without clearly visible vascularity, and could therefore be considered a marker for early AHM.

## FUNDING INFORMATION

The Italian Ministry of Health (Ricerca Corrente) partially supported this work.

## CONFLICT OF INTEREST STATEMENT

None declared.
